# *Buchanania obovata*: Functionality and Phytochemical Profiling of the Australian Native Green Plum

**DOI:** 10.3390/foods7050071

**Published:** 2018-05-04

**Authors:** Selina A. Fyfe, Gabriele Netzel, Michael E. Netzel, Yasmina Sultanbawa

**Affiliations:** 1 School of Agriculture and Food Sciences, The University of Queensland, St Lucia, Brisbane, QLD 4072 Australia; selina.fyfe@uqconnect.edu.au; 2 Queensland Alliance for Agriculture and Food Innovation (QAAFI), The University of Queensland, Health and Food Sciences Precinct, 39 Kessels Rd Coopers Plain;, PO Box 156, Archerfield, QLD 4108, Australia; g.netzel@uq.edu.au (G.N.); m.netzel@uq.edu.au (M.E.N.)

**Keywords:** *Buchanania obovata*, green plum, fruit, polyphenols, antioxidants, Indigenous Australia

## Abstract

The green plum is the fruit of *Buchanania obovata* Engl. and is an Australian Indigenous bush food. Very little study has been done on the green plum, so this is an initial screening study of the functional properties and phytochemical profile found in the flesh and seed. The flesh was shown to have antimicrobial properties effective against gram negative (*Escherichia coli* 9001—NCTC) and gram positive (*Staphylococcus aureus* 6571—NCTC) bacteria. Scanning electron microscopy analysis shows that the antimicrobial activity causes cell wall disintegration and cytoplasmic leakage in both bacteria. Antioxidant 2,2-diphenyl-1-picrylhydrazyl (DPPH) testing shows the flesh has high radical scavenging activity (106.3 ± 28.6 μM Trolox equivalant/g Dry Weight in methanol). The flesh and seed contain a range of polyphenols including gallic acid, ellagic acid, p-coumaric acid, kaempferol, quercetin and trans-ferulic acid that may be responsible for this activity. The seed is eaten as a bush food and contains a delphinidin-based anthocyanin. The green plum has potential as a functional ingredient in food products for its antimicrobial and antioxidant activity, and further investigation into its bioactivity, chemical composition and potential applications in different food products is warranted.

## 1. Introduction

*Buchanania obovata* Engl. is a native Australian tree that grows in the northern parts of Australia in Western Australia and the Northern Territory [1]. It produces a small green fruit known as the green plum which is eaten by Indigenous Australians. As a food it is eaten straight from the tree or with the flesh and seeds mashed into a paste, and is a favourite with children [2,3].

The *B. obovata* plant is in the family Anacardiaceae, which also contains the mango (*Mangifera indica*), cashew apple (*Anacardium occidentale*) and pistachio nut (*Pistacia vera*) [4].

The green plum was selected to be in this study because it is commonly eaten as a food and parts of the tree are used as bush medicine by Australian Aboriginal people. Leaf ribs, young stems, and the inner bark from young branches and older stems are used as bush medicine for their antiseptic and analgesic qualities to treat toothache, skin conditions and infections, and as an eye lotion [5,6].

Other native Australian foods have good nutritional and functional properties. The Kakadu plum (*Terminalia ferdinandiana*) has very high levels of ascorbic acid, high antioxidant capacity [7,8], and strong antimicrobial activity, enabling it to be used as a natural preservative for the commercial dipping of prawns in Queensland, Australia [9]. It is increasingly being used for its functional properties and is currently used in food and beauty products [10]. Davidsons plum (*Davidsonia pruriens* F. Muelli) has shown vitro anti-proliferative activity against a variety of cancer cells [11] and can be used as a natural preservative in meat [9].

Synthetic antioxidants like butylated hydroxytoluene (BHT), butylated hydroxyanisole (BHA) and propyl gallate are commonly used in food products, but have been associated with carcinogenic and toxic effects [12]. Risk perceptions of chemicals in foods cause people to prefer natural foods [13]. A total of 84% of people in the 2017 Eurobarometer survey said they are worried about the impact of chemicals present in everyday products on their health [14]. The food industry is increasingly trying to replace synthetic antioxidants with natural ingredients that are safer. There is interest in the use of foods that inherently contain bioactive compounds with these properties and can be used as food additives and ingredients [15].

This study seeks to determine the functional properties (antimicrobial and antioxidant) of the green plum flesh and seed and the components that give it these properties. This is the first study of this kind on the green plum and will give an understanding for its potential use as both a food and as a functional ingredient in food processing.

## 2. Materials and Methods 

### 2.1. Chemicals

The chemicals used were from Sigma-Aldrich (Castle Hill, NSW, Australia). The Standard Plate Count Agar (APHA) CM0463 was from Oxoid Ltd., (Basingstroke, England).

### 2.2. Sample Preparation

The *B. obovata* fruit were hand-picked fresh at a maturity stage that was slightly under-ripe to optimise the phytochemicals and for ease of transportation. They were picked from trees near Darwin, Northern Territory, Australia, in 2015. The whole fruit were frozen, transported to Brisbane and remained frozen at −20 °C. A total of 554 g of the whole fruit with seeds intact (approximately 700 green plums) were lyophilised in a Christ Gamma 1-16 LSC Freeze Drying Unit (John Morris Scientific, Osterode, Germany), then flesh and seed were separated by blending the flesh to a powder in a kitchen food processor. The seeds were milled into a powder using a hammer mill (Lab Mill, Christy and Norris Ltd., Chelmsford, England). The composite flesh and seed powders were stored separately at room temperature, protected from light and in air-tight plastic containers.

### 2.3. Accelerated Solvent Extractions

Extractions were carried out using an Accelerated Solvent Extractor (ASE) (ASE 350, Dionex Corporation, Sunnyvale, CA, USA) with a method slightly modified from that in Navarro et al. [16]. Briefly, extractions were performed on 1 g samples of flesh powder or seed powder in triplicate using 100% methanol, 95% ethanol or distilled water as solvents, at 60 °C, 60 °C and 80 °C respectively with eight cycles. The extractions were filtered then evaporated until dry in a miVac evaporator (Genevac Ltd., Ipswich, England). The resulting extract powder was stored in air tight plastic containers at −20 °C.

### 2.4. Extraction of Unbound Polyphenols

The unbound polyphenol extraction method was slightly modified from Kammerer et al. [17]. Samples of 200 mg of flesh and seed powder were extracted in triplicate with methanol:water:hydrochloric acid (80:19:1) three times on a reciprocating shaker (RP1812, Paton Scientific, Stepney, SA, Australia) at 200 rpm for 2 h at ambient temperature, then centrifuged and the supernatant was removed. The supernatant and residue were stored separately at −20 °C.

### 2.5. Extraction of Bound Polyphenols

The extraction of the bound polyphenols was modified from Adom and Liu [18]. The residue from the unbound polyphenol extraction was hydrolysed for 1 h at 200 rpm in 2 M NaOH on a reciprocating shaker at ambient temperature. It was acidified to pH 2.0 with concentrated HCl and extracted five times with ethyl acetate. The ethyl acetate layers were dried under nitrogen at 40 °C (Dry Block Heater, Ratek Instruments, Boronia, VIC, Australia) before being reconstituted in 50% methanol and stored at −20 °C.

### 2.6. In Vitro Antioxidant Capacity

ASE extraction aliquots were made up in their extraction solvent to 1 mg/mL with dilutions, and tests were done in triplicate from the triplicate extracts of each solvent (*n* = 9). Tests were done in 96-well plates and read on a Tecan Microplate Reader (Tecan Infinite M200, Tecan Trading AG, Mannedorf, Switzerland) with Magellan Software (version 6.4, Tecan Trading AG, Mannedorf, Switzerland).

The total phenolics content (TPC) measured the reducing capacity of the flesh and seeds and was modified from Singleton and Rossi [19] and Ahmed et al. [20] with gallic acid standards on all extractions. Results are presented as g gallic acid equivalents (GAE)/kg dry weight (DW).

Radical scavenging activity was measured using a 2,2-diphenyl-1-picrylhydrazyl (DPPH) method modified from Yu and Moore [21], with Trolox standards at a concentration range of 5–35 µM/L. Briefly, equal amounts of control, standard or samples and 0.15 mM DPPH were mixed, incubated, and the absorbance read at 517 nm. Results were calculated to µM Trolox equivalents (TE)/g DW.

Chelating activity was measured using a ferrous ion chelating (FIC) assay modified from Decker and Welch [22], Kuda et al. [23] and Wang et al. [24], with ethylenediaminetetraacetic acid (EDTA) as a positive control. Briefly, 200 μL of solvent controls or samples and 10 μL of 1 mM ferrous chloride were mixed and the absorbance read at 562 nm to obtain blank results. 15 μL of 2.5 mM ferrozine was added, it was incubated (10 min, dark, ambient temperature), and the absorbance read at 562 nm. The blank was subtracted from the result reading and % chelating was calculated using the solvent control.

### 2.7. Antimicrobial Activity

Antimicrobial activity was tested on each type of ASE extraction using a Kirby–Bauer disc diffusion assay modified from Dussault et al. [25]. Standard Plate Count Agar (APHA CM0463, Oxoid Ltd., Hampshire, England) was spread with either *Staphyloccocus aureus* strain 6571 (NCTC—National Collection of Type Cultures, Health Protection Agency Centre for Infection, London, UK) or *Escherichia coli* strain 9001 (NCTC) at a McFarland turbidity of absorbance 0.1 at 540 nm. Triplicates of 75 µL of concentrated extract in 20% ethanol were added to aseptically placed discs on the bacteria coated agar, with 20% ethanol as the control. The concentration of extract on each disc was equivalent to approximately 130 mg DW of flesh freeze dried powder and 60 mg DW of seed freeze dried powder. Plates were incubated (Sanyo Incubator, MIR-154, Sanyo Electric Co., Ltd., Osaka, Japan) at 37 °C for 24 h. The diameter of the zone of inhibition was measured in mm under a lighted magnifying glass using a 150 mm digital caliper (Craftright Engineering Works, Jiangsu, China).

### 2.8. Scanning Electron Microscopy of Antimicrobial Activity

The same *S. aureus* and *E. coli* strains were grown for 7 h in tryptone soy yeast extract broth (TSYEB) at 37 °C. A total of 75 µL of concentrated green plum flesh water extract in 20% ethanol (from approximately 130 mg DW of flesh powder) was added to 1 mL bacteria and broth samples, and 75 µL of 20% ethanol was added to the controls, and incubated for 24 h at 37 °C. The samples and controls were washed three times in sterile phosphate buffered saline and fixed in 3% glutaraldehyde. They were adhered to poly-l-lysine-coated (1 mg/mL) coverslips and dehydrated in ethanol before being dried in a critical point dryer (Tousimis Research Corporation, Rockville, MD, USA). Coverslips were attached to stubs with double-sided carbon tabs and coated with gold before samples were imaged using scanning electron microscopy (SEM) on a Jeol Neoscope JCM 5000 (Jeol Ltd., Tokyo, Japan) at an accelerating voltage of 10 kV.

### 2.9. Phytochemical Quantification and Identification

The phytochemicals in the extracts of the free and bound polyphenols as well as the ASE extracts were quantified by ultra pressure liquid chromatography—photo diode array detector (UPLC-PDA) following a modified method of Gasperotti et al. [26]. The compounds were separated on a Waters Acquity HSS T3 (100 × 2.1 mm; 1.8 μm) column at 40 °C using the following gradient: 5–20%B (3 min), isocratic (1.3 min), 20–45%B (4.7 min), and 45–100%B (2 min). Mobile phase A consisted of water and mobile phase B of acetonitrile, both containing 0.1% formic acid. The flow was set to 0.4 mL/min and 5 µL of the pre-filtered sample (0.2 μm, Pall, Cheltenhan, VIC, Australia) was injected into the system. The compounds were quantified using external calibrations of the individual phenolic compounds and the peak identity was confirmed by LC-MS.

The same method as described above was used on a ultra high pressure liquid chromatography—mass spectrometry (UHPLC-MS) QExactive (Thermo Fisher Scientific, Bremen, Germany) for peak identification. The LCMS was used in the negative mode, and were run against 39 different standards as a screening study. The seed unbound extraction had anthocyanin identified on a UHPLC-MS QExactive in the positive mode and quantified on a UHPLC with a PDA detector (Agilent, Wilmington, DE, USA).

### 2.10. Statistical Analysis

Data analysis and the calculations of results was carried out using Microsoft Excel software, version 2013 (Microsoft Corporation, Redmond, WA, USA) and Minitab 16 Statistical Software (Minitab Inc., State College, PA, USA). Data is presented as arithmetic means ± standard deviations.

## 3. Results and Discussion

### 3.1. In Vitro Antioxidant Capacity

The in vitro antioxidant capacity of the flesh and seed are given in Table 1. In all three tests, the methanol extractions gave higher results than the ethanol extractions, which were higher than the water extractions apart from the FIC on the seed. The percent radical scavenging activity of the green plum flesh and seed was measured at a concentration of 1 mg/mL of extract powders, and the flesh gave 93.6 ± 0.6% in methanol, 92.5 ± 0.6% in ethanol and 92.7 ± 1.0% in water. The seed had similarly high levels at the same concentration of extract powder with methanol 93.2 ± 0.3%, ethanol 92.4 ± 0.7% and water 90.8 ± 1.3%. These levels are similar to that of the Kakadu plum, and higher than the Australian native wild lime, finger lime and almost twice that of the Davidson’s plum in non-acidified extracts [27]. The radical scavenging activity is much higher than the chelating ability in both the flesh and seed at the same concentration, which could affect its application in food products.

The TPC of the unbound extraction was eight times higher than the Australian desert lime and lemon aspen, nearly three times higher than the quandong and riberry and one and a half times higher than both types of Davidson’s plum studied by Konczak et al. [28]. It was lower than the Kakadu plum (approx. 158 g GAE/kg DW) but over twice the content of the blueberry control [28].

### 3.2. Antimicrobial Activity

**Figure 1:**
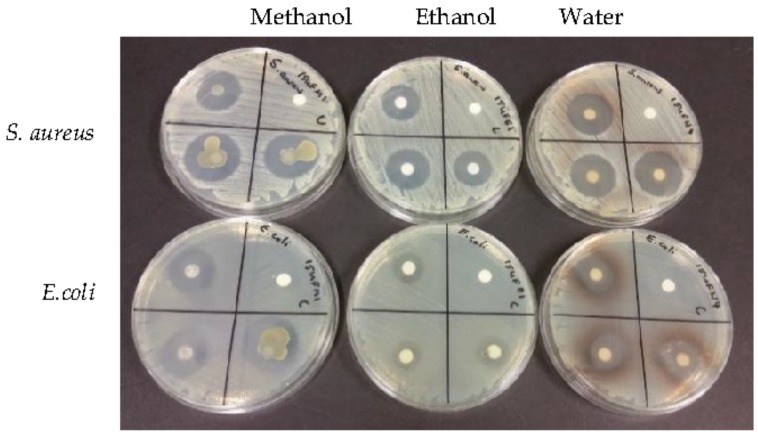
Disc diffusion assay of flesh against top row, *S. aureus* and bottom row *E. coli*, from left to right extracts are methanol, ethanol and water, with controls in top right hand corner of plates. *n* = 3.

Table 2 shows the antimicrobial results of the disc diffusion assay and Figure 1 shows the flesh results.

The flesh results show that it has much higher antimicrobial activity than the seed. The flesh inhibited the growth of bacteria in all extracts for both types of bacteria. The seed only gave a low amount of inhibition for the gram positive *S. aureus* and none for the *E. coli*. Aboriginal Australians use parts of *B. obovata* plant as bush medicine for its antimicrobial properties and this work adds to the growth inhibition found by Barr et al. [6] for the leaves and twigs. Figure 1 shows that in the water extractions the flesh both inhibited growth and also appears to have turned them a brown colour.

**Figure 2:**
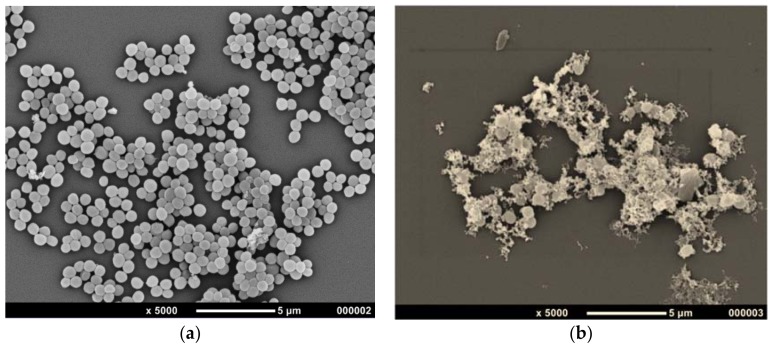

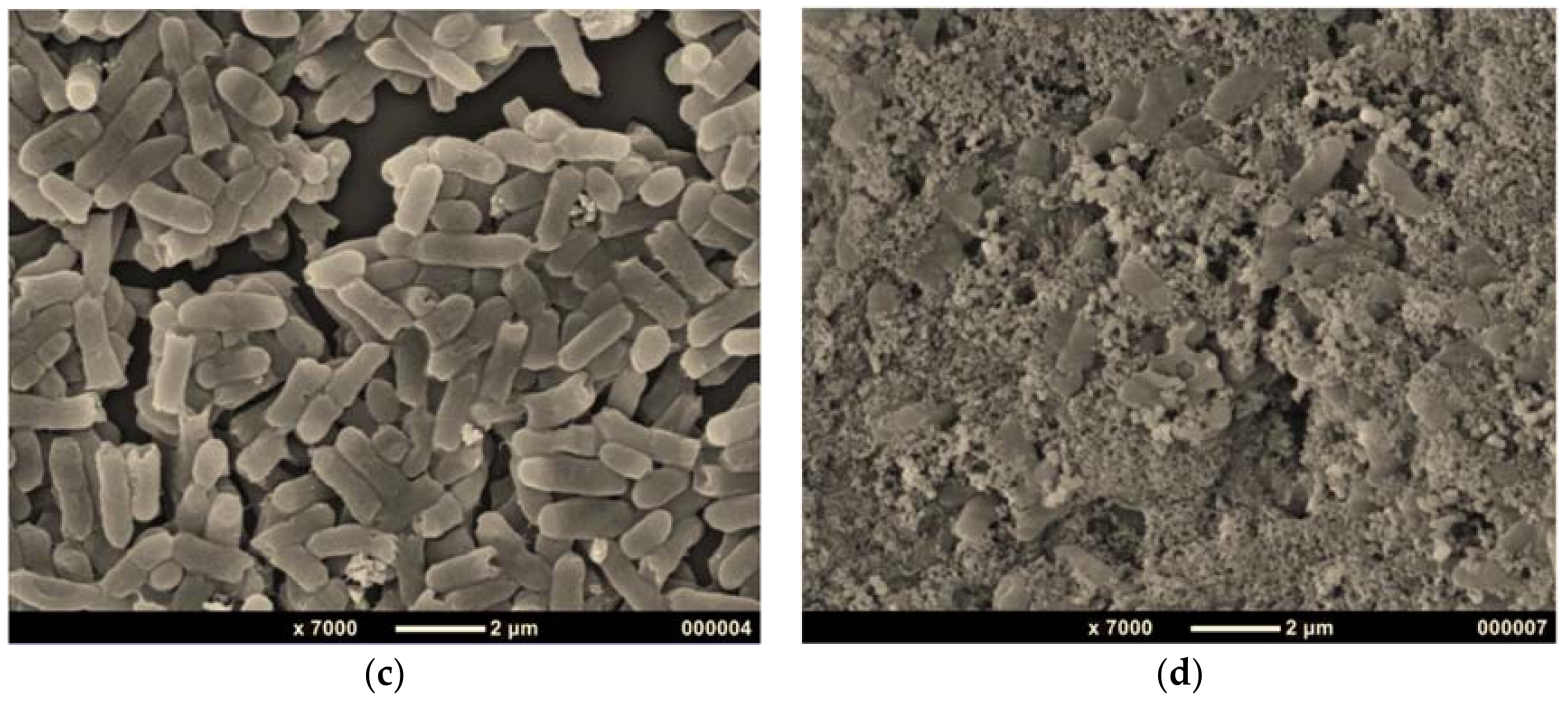
Scanning electron microscopy images of (**a**) *S. aureus* control; (**b**) *S. aureus* with treatment from green plum flesh water extract; (**c**) *E. coli* control and (**d**) *E. coli* with treatment from green plum flesh water extract.

Further investigation of the antimicrobial properties was carried out using SEM imaging on the same bacteria types and the water extraction of the flesh. Figure 2 shows the SEM images of the controls and green plum flesh water extract treatment of *S. aureus* and *E. coli*.

The *S. aureus* control cells showed smooth round surfaces and membrane integrity. The treated *S. aureus* showed shape changes, with misshapen cells, broken open cells, swelling and changes to the cell structure. There was significant leakage of cytoplasmic inclusions and some cells showed this occurring. There were cells with changes to their morphology with deep wrinkles in them, as well as indentations and changes to their size from swelling. One had clearly broken open and the cell wall opened out, and others appeared to be broken bits of cell wall. Although there were a few cells that appeared more intact, their numbers were much lower than in the control. The *E. coli* control cells generally displayed smooth surfaces with occasional surface pitting and possible cell death from 31 h of growing. In the image from the treatment there is evidence of complete cell collapse, pitting and breaking in the side wall of the cells, misshapen cells and leakage of cytoplasmic components. These indicate that there was an almost complete collapse of the cell structure, which was accompanied by cell lysis. The images show that the flesh extract in water does have antimicrobial activity. Cell disintegration may be what caused the dark colour in the flesh water extract disc diffusion plate.

These images show changes that are similar to those shown by Zhang et al. [29] in their study on the antimicrobial activity of D-limonene. The deformation and distortion caused by the green plum extract indicates strong antimicrobial activity on the cellular integrity, similar to that shown by the combined treatment of D-limonene nanoemulsions with nisin [29].

### 3.3. Phytochemical Analysis

The analysis of polyphenols in the green plum flesh and seed powders were carried out as a screening study of 39 different polyphenols chosen for their presence and prominence in fruit and in the mango, to which the green plum is related. The compounds identified in the various extracts are shown in Table 3. The compounds which had concentrations high enough to be quantified by UPLC-PDA are shown in Table 4.

The green plum flesh and seed contain a number of compounds with antioxidant and antimicrobial activity as well as other beneficial properties. The high radical scavenging ability shown in the DPPH assay may be due to the presence of gallic acid, which was present in all extractions. Gallic acid is able to scavenge a variety of free radicals including singlet oxygen, hydroxyl, peroxyl and alkyl peroxyl and protects against UV cell damage [30]. *Trans*-ferulic acid is a low toxic component of plants and protects against pathogen invasion and has antioxidant, antimicrobial and anti-inflammatory activities [31]. It scavenges reactive oxygen species and reactive nitrogen species and nanoparticles containing it reduce lipid peroxidation [32].

Chlorogenic acid inhibits a range of bacterial pathogens by increasing outer membrane permeability, inducing the efflux of potassium from the cell, and causing rupture of the membrane and leakage of the cytoplasmic contents, including nucleotides [33].

Some flavonoid combinations can work synergistically in their antioxidant capacity, including kaempferol and quercetin (+19.9%) and catechin and kaempferol (+2.4%) [34], all found in the green plum flesh. Catechin and quercetin may mitigate adipose inflammation, oxidative stress and insulin resistance and have protective effects on human health [35]. They are antioxidants with radical scavenging activity and reducing properties [36] and quercetin is active against oxidation deterioration in emulsions [15].

Naringenin was also found and it has antioxidant, anti-carcinogenic and anti-inflammatory effects [37]. Ellagic acid has antioxidant functions and has an inhibitory effect on the growth of micro-organisms [38]. There is an abundance of ellagic acid in the Kakadu plum that contributes to its use as a natural food preservative [7], and it was found in both the flesh and seed of the green plum.

The unbound seed extractions were pink in colour and was analysed for anthocyanin. The spectrum was characteristic for anthocyanin compounds and the associated fragments in the LC-MS showed an *m*/*z* of 303.0482 and a structure of C_15_H_11_O_7_, which is indicative of a delphinidin aglycone. The quantification was done against a cyanidin 3-glucoside calibration curve and the concentration of the anthocyanin was 423 ± 8.04 µg cyanidin 3-glucoside equivalents/g DW in the seed. Anthocyanins have been found previously in the seed coats of common beans using a similar extraction method [39], and in the seed coats of black soybeans, including delphinidin-3-glucoside [40].

Antioxidants are important in food to prevent the oxidation that occurs naturally and causes off-flavours and rancidity, loss of fat-soluble vitamins, fatty acids and bioactives, and can sometimes form toxic compounds [41]. There is an increased interest from consumers and the food industry in replacing synthetic preservatives with natural ones, such as plant material that inherently has these properties [15]. Other Australian native plant foods are being incorporated into food products for their natural preservative effects [9]. Plant extracts can be used in the food industry to increase the shelf-life of food products, using their natural antioxidant and antimicrobial properties to control the growth of food-borne pathogens [42]. The green plum could potentially be used as a natural alternative to chemical additives to increase the shelf life and quality of food. The antioxidant and antimicrobial potential of the green plum indicate it may be able to be used as a functional ingredient.

## 4. Conclusions

The green plum has considerable antimicrobial activity in its flesh, the water extraction caused bacterial cell deformation and disintegration. The radical scavenging activity is high particularly in the flesh. These activities may be the result of a number of the phytochemicals found in its flesh and seed including gallic acid, ellagic acid, p-coumaric acid, kaempferol, trans-ferulic acid and quercetin. The seed was also found to contain anthocyanins that have yet to be identified completely. The green plum is an Australian native fruit that has potential for use as a food preservative and further investigation into these uses is justified.

## Figures and Tables

**Table 1:** In vitro antioxidant capacity of green plum flesh and seed freeze dried powders.

Extraction	TPC Flesh g GAE/kg DW	TPC Seed g GAE/kg DW	DPPH Flesh µM TE/g DW	DPPH Seed µM TE/g DW	FIC Flesh * % Chelating	FIC Seed * % Chelating
**Methanol**	19.2 ± 4.4 ^b^	2.6 ± 0.6 ^b^	106.3 ± 28.6 ^a^	14.6 ± 3.6 ^a^	21.4 ± 8.9 ^a^	20.8 ± 9.4 ^b^
**Ethanol**	10.3 ± 3.0 ^c^	N/A	40.2 ± 13.4 ^b^	10.0 ± 3.8 ^b^	4.9 ± 6.4 ^b^	17.8 ± 16.6 ^b^
**Water**	5.0 ± 1.6 ^d^	1.4 ± 0.2 ^c^	34.7 ± 13.4 ^b^	11.5 ± 2.8 ^a,b^	3.5 ± 5.2 ^b^	34.9 ± 7.3 ^a^
**Unbound**	84.6 ± 5.3 ^a^	6.5 ± 0.5 ^a^	N/A	N/A	N/A	N/A
**Bound**	9.2 ± 2.8 ^c,d^	1.1 ± 0.2 ^c^	N/A	N/A	N/A	N/A

*n* = 9; * FIC % Chelating at concentration of 1 mg/mL of extract; N/A not available, mean values of each column with a different alphabet letter are significantly different (at *p* < 0.05).

**Table 2:** Antimicrobial activity by disc diffusion assay of the green plum flesh and seed, results as diameter of inhibition zone in mm using a 6 mm disc.

Extraction	Flesh *E. coli* (mm)	Flesh *S. aureus* (mm)	Seed *E. coli* (mm)	Seed *S. aureus* (mm)
**Methanol**	20.73 ± 1.27 ^a^	22.61 ± 1.42 ^a^	NI	8.83 ± 0.24 ^a^
**Ethanol**	9.55 ± 2.40 ^c^	20.38 ± 1.14 ^b^	NI	NI
**Water**	16.48 ± 0.95 ^b^	23.44 ± 0.85 ^a^	NI	6.81 ± 0.10 ^b^

*n* = 3 All controls had no inhibition. NI: No inhibition observed. Mean values of each column with a different alphabet letter are significantly different (at *p* < 0.05).

**Table 3:** Compounds found in green plum flesh and seed extracts using ultra high pressure liquid chromatography—mass spectrometry (UHPLC-MS) QExactive in negative mode.

		Flesh					Seed				
	*m*/*z*	U	B	M	E	W	U	B	M	E	W
*Trans*-ferulic acid	193.0506	X	X	X	X	X		X	X	X	X
p-coumaric acid	163.0401	X	X	X	X	X	X		X	X	X
hydroxybenzoic acid	137.0244						X	X	X	X	X
Salicylic acid	137.0244			[x]	[x]	X					
Catechin	289.0718	X		X	X	X	X	X	X		X
Gallic acid	169.0143	X	X	X	X	X	X	X	X	X	X
Kaempferol	285.0405	X		X	X						
Naringenin	271.0612	X	X	X	X		X		X	X	
Quercetin	301.0354	X		X	X	X	X		X	X	[x]
Quercetin 3-glucoside	463.0882	X	X	X	X	X	X	X	X	X	X
Quercetin 3 rutinoside	609.1461	X	X	X	X	X	X		X	X	X
Quercetin 3-xyloside	433.0776	X	X	X	X	X			X	X	[x]
Chlorogenic acid	353.0878	X		[x]			[x]		X	X	
Cinnamic acid	147.0452	X									
Vanillic acid	167.0350						X				
Isorhamnetin-3-glucoside	477.1039	[x]		[x]	[x]	[x]	X		X	X	
Quercetin 3,4′-diglucoside	625.1410	X		X	X	X	X		[x]	X	X
Procyanidin B1	577.1352	[x]		[x]	[x]	[x]			X		[x]
Eriodicyol 7-glucoside	449.1089	X		X	X	X			X	X	X
Ellagic acid	300.9990		X	X	X	X	X	X	X	X	X

X, compounds present; [x], compound present in trace amounts; U, unbound; B, bound; M, methanol; E, ethanol; W, water. *n* = 1.

**Table 4:** Quantified phytochemicals in green plum flesh and seed unbound and bound extractions.

	Flesh Unbound (μg/g DW)	Flesh Bound (μg/g DW)	Seed Unbound (μg/g DW)	Seed Bound (μg/g DW)
Gallic Acid	955.39	3342.97	151.89	3.95
Myricetin	180.96			
Quercetin 3,4′-diglucoside				63.37
Chlorogenic acid	91.83	19.93	695.19	45.71
Ferulic Acid	114.50	8.02	22.77	17.85
p-coumaric acid			4.42	
Quercetin 3-glucoside	874.17	39.64	13.19	
Quercetin	118.59			
